# Towards Improvements for Penetrating the Blood–Brain Barrier—Recent Progress from a Material and Pharmaceutical Perspective

**DOI:** 10.3390/cells7040024

**Published:** 2018-03-23

**Authors:** Quanguo He, Jun Liu, Jing Liang, Xiaopeng Liu, Wen Li, Zhi Liu, Ziyu Ding, Du Tuo

**Affiliations:** School of Life Science and Chemistry, Hunan University of Technology, Zhuzhou 412007, China; hequanguo@126.com (Q.H.); liu.jun.1015@163.com (J.L.); liangjingabbey@126.com (J.L.); amituo321@163.com (X.L.); liuzhi0910@163.com (Z.L.); dingziyu0320@163.com (Z.D.); dutuo99@hut.edu.cn (D.T.)

**Keywords:** blood–brain barrier, drug delivery system to brain, central nervous system, diseases, permeability improvements

## Abstract

The blood–brain barrier (BBB) is a critical biological structure that prevents damage to the brain and maintains its bathing microenvironment. However, this barrier is also the obstacle to deliver beneficial drugs to treat CNS (central nervous system) diseases. Many efforts have been made for improvement of delivering drugs across the BBB in recent years to treat CNS diseases. In this review, the anatomical and functional structure of the BBB is comprehensively discussed. The mechanisms of BBB penetration are summarized, and the methods and effects on increasing BBB permeability are investigated in detail. It also elaborates on the physical, chemical, biological and nanocarrier aspects to improve drug delivery penetration to the brain and introduces some specific drug delivery effects on BBB permeability.

## 1. Introduction

The blood–brain barrier (BBB) is the complex network of brain microvessels. It protects the brain from the external bloodstream environment and supplies the brain with the required nutrients for normal function. Unlike the peripheral capillaries that allow the relatively free exchange of substances between blood and tissues, the BBB has the least permeable capillaries in the entire body due to physical barriers (tight junctions). Thus, the BBB is a typically rate-limiting factor for the availability of therapeutic drugs that penetrate the brain. Therefore, it is crucial to discover delivery systems which can cross this barrier for the treatment of brain-based diseases, such as Alzheimer’s disease (AD), Parkinson’s disease (PD) and epilepsy. Many researchers were dedicated to addressing this challenge, and a number of methods were developed to improve BBB permeability via in vivo, in vitro and in silico methods. In this review, the tissue and functional structures of the BBB were discussed comprehensively, and the methods and mechanisms for increasing penetration of the BBB were summarized in detail.

## 2. The Discovery and Structure of BBB

In late 1600, the first notions of specific protective nature of the brain were being described by Humphrey Ridley. In addition, Lewandowki also performed some studies in animals in the early 1900s and suggested that brain capillaries must hold back certain molecules [1]. Over 100 years ago, Paul Ehrlich discovered a vascular barrier between the blood circulation and the central nervous system (CNS). He found that some dyes were rapidly dispersed by all organs except brain and spinal cord when injected into the vascular system. Ehrlich explained these phenomena as a lack of affinity of these dyes to the cerebral vascular endothelium. Shortly afterward, however, Edwin E. Goldman found out that some dyes can only selectively stain the nervous tissue when they were injected into the cerebrospinal fluid [2], meaning that these dyes were prevented from entering the blood circulation of the brain. Thereafter, the concept of a vascular blood–brain barrier, which also functions as a brain–blood barrier was put forward. Then, the “blood–brain barrier” (BBB) began to be recognized [3].

**Figure 1:**
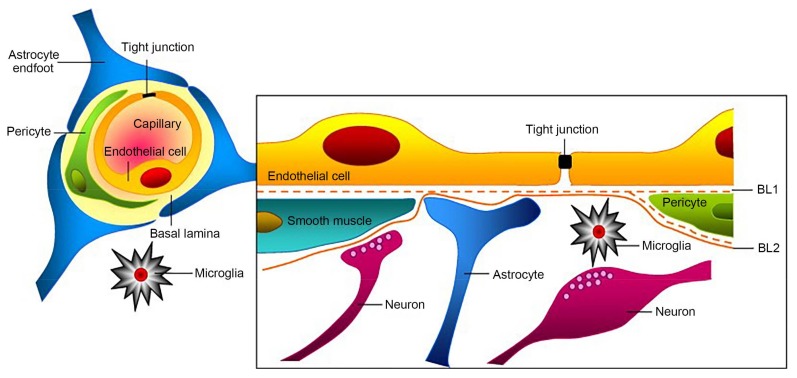
Schematic representation of the structure of blood–brain barrier (BBB) (BL1 is basal lamina 1, BL2 is basal lamina 2). Reprinted with permission from ref. [11].

The BBB acts as a strict control point for what can enter the brain and regulate CNS internal milieu, which is created by tight junctions (TJs) between endothelial cells (ECs) lining blood vessels, astroyctic endfeet, and a basement membrane [4,5]. Capillaries are the smallest cerebral blood vessels and they account for approximately 85% of cerebral vessel length and are a major site of the blood brain barrier as shown in Figure 1. Among them, ECs and TJs are the basic structures in the BBB. TJs are the complicated cell line with regulating effect, and it significantly reduces the permeation of ions and other small hydrophilic solutes by paracellular pathway, which is a physical barrier [6,7,8,9]. Other essential molecules pass into the brain by transcellular pathways predominantly (usually active transporters). The tight junctions consist of complex proteins spanning the intercellular cleft, including the transmembrane proteins, cytoplasmic attachment proteins, and cytoskeletal proteins. Transmembrane proteins are composed of occluding, claudins and junctional adhesion molecules (JAMs). Cytoplasmic attachment proteins are constituted three cingulin ZO-1, ZO-2, ZO-3. The expressions and arrangements of these proteins are closely related to the function of BBB. Some drugs or pretreatments can affect the expression of TJs proteins and influence the expression of BBB permeability indirectly.

Moreover, the efflux effect of P-glycoprotein (P-gp) exists on the surface of ECs. These proteins are a kind of matrix extracellular phosphoglycoproteins (MEPE), which are dependent on the transfer of adenosine triphosphate (ATP) [10]. Under the function of ATP, P-glycoprotein makes the inverse concentration of toxicant transfer from intracellular to extracellular for decreasing the intracellular drug concentration. At the same time, the expression efficiency of P-gp is 400–500 higher than meninx. The high expression of P-gp is one of the important mechanisms of multiple drug resistance (MDR) in cancer therapy, and it is also one of the main reasons that a lot of lipophilic drugs could not penetrate into CNS for brain disease therapy. The development of P-gp inhibitor is the main method for cancer therapy, which has been researched in clinical aspect for several decades. The effective inhibitor could prevent the P-gp to efflux effect. Moreover, finding the metabolic pathways of P-gp in the human body is another way for changing the functional mechanism of it, and drugs could play its full role in disease therapy. Furthermore, several efflux transporters, such as the breast cancer resistance protein (BCRP), glucose transporter (GLUT), multidrug resistance proteins (MRP) and the BBB choline transport (BBB-ChT), have profound clinical relevance to several CNS diseases. Although the macromolecules are not allowed to pass through the BBB, the small molecule can still penetrate the BBB via unusual transporters of endothelial cell. The BBB not only possesses the anatomical structure but also shows the functional structure of the barrier and selection specification.

## 3. Research Progress in Methods for Blood–Brain Barrier Permeability and Circumvention

With existence of the BBB, many macromolecules could not enter the brain. Only some micromolecule (molecular weight < 600 Da, chain length < 6 amino acid) and lipid soluble molecules could across the BBB and then arrive passively to the brain. The BBB restricts the penetration into the brain, not only of large-molecule drugs, but also of more than 98% of small-molecule drugs, such as the anticancer drugs paclitaxel, adriamycin, methotrexate, and vincristine [12]. In the past, various techniques have been exploited to pass through the BBB, including transcranial or nasal administration, infused hypertonic agents, and lipidation of small-molecule drugs. The specific information is depicted in Table 1.

However, these methods have some limitations as most of them are invasive, and they could lead to the risks of infection or brain trauma if these methods were employed, and they may not be amenable for repeated treatments or drug delivery to large areas of the brain. Also, these methods usually cause delivery of drugs to white matter which is often not desirable. Recently, some newly methods were investigated in improving BBB permeability to or overcoming some undesirable results and side effect. These methods are versatile and some of them are substantially available and noteworthy, the strategies include the following areas: physical, chemical, biological and various nanoparticle systems.

### 3.1. Physical Methods 

Focused ultrasound (FUS) is used in contrast-enhanced magnetic resonance imaging (MRI) in the early outset because of its good resolution in time and space. Using this technique in the medical field could acquire the diagnostic message without tissue damage. In Jenne and Kraffts’s study, contrast-enhanced MRI signal changed after a FUS-mediated BBB opening is quantified for two different types of MRI contrast agents [26]. In particular, the short-term signal changes of the interstitial magnevist contrast agent demonstrate an immediate signal enhancement. Hence, an instantaneous BBB opening could obtain with the incipient FUS administration and a US contrast agent. With the aid of combined focused ultrasound (FUS) with a circulating microbubble agent, the BBB could be opened temporarily. This method is a localized and noninvasive emerging technique that facilitates the permeability of BBB.

In the 1950s, the permeability of BBB is found to be enhanced by the FUS. Scholars found that the BBB could be selectively and reversibly opened by the microbubbles interacted with an ultrasound field application. The opened time is prolonging to 4 h, and there were no evident changes of neurobehavior or morphological changes of brain tissues in mice. These findings were provided as the reference for therapy of CNS diseases. Compared with the common methods in the clinic (such as hyperosmotic glucose), this method shows some advantages in opening the BBB, such as being noninvasive, having a good selectivity of opening position, no obvious side effects, and reusable. However, as shown in the early research, the bleeding or heat coagulation phenomena are observed along with the BBB opening. The heat injury is found in the area of BBB permeation, and the threshold is related to the tissue damage of BBB. Generally, the bleeding and heat coagulation phenomena are influenced by pulse duration, pulse count, and repetition frequency closely. The specific information is shown in Table 2. Nevertheless, the BBB is still opened by this method. However, the biological effect of this process as well as the in silico simulation needs further research [27,28].

Recently, Polat et al. used microbubble-associated low-frequency sonophoresis (LFS) (42.6 kHz) method to induce the apoptosis of vascular smooth muscle cells for achieving changes in cell permeability. Specifically, reducing the expression of a protein related to TJs and changing the distribution of it could open the blood–tumor barrier. This method was used in the clinic for the delivery of liposomal lidocaine, or lidocaine/prilocaine (both of them were small and hydrophobic molecules). The onset time for local anesthesia was decreased correspondingly. This application had been well-documented, as multiple pilots and clinical trials have shown that LFS decreased the onset to anesthesia with lidocaine, from 30–60 min passively to less than 5 min with LFS pretreatment. LFS technology was currently food and drug administration approved for use in local anesthetics [29]. Recent work demonstrates that the safety of a clinical brain FUS system is proved by a large number of animal models. This result indicates that the FUS system is a promising approach for the treatment of brain diseases [30].

In general, opening is a noninvasive targeted behavior, and could not damage the brain. The method was also compatible with currently available drugs, removing the need to develop new agents. Importantly, since FUS was presumably noninvasive without general anesthesia, it was expected to be a relatively benign procedure that could be readily repeated to match a patient’s drug schedule. However, this method can lead to endothelial cell damage in the long-term, which can lead to the opening of the blood–brain barrier temporarily. Moreover, long time ultrasound can be harmful to BBB. In general, the FUS technique to enhance the drug delivery across the BBB to the brain still has a pronounced therapeutic effect in vitro and animal model.

### 3.2. Chemical Methods

Using chemical methods to strengthen the penetration properties of BBB are a new way and also a possible alternative to improve the therapeutic effect of brain disease, which is carried out by using the chemical technique to modify and endow the drugs with the lipotropic surface. As discussed above, BBB is one of the limiting factors for the drug delivery treatment of brain diseases and cancers. A very restricted number of liposoluble small molecules (MW < 400 Da) could cross the BBB by free diffusion. The current chemical methods for crossing the BBB are mainly divided into three types: (1) chemical modification of the drug to form a prodrug, which is usually more lipophilic than parent agents; (2) coupling the drugs with mannitol or aromatic substances (such as borneol and musk), because mannitol could induce a high osmotic pressure to opening the BBB temporarily and aromatic substances could cross the BBB as the resuscitation medicine; (3) using appropriate chemical drug delivery system or drug carrier with the ability to cross BBB.

**Figure 2:**
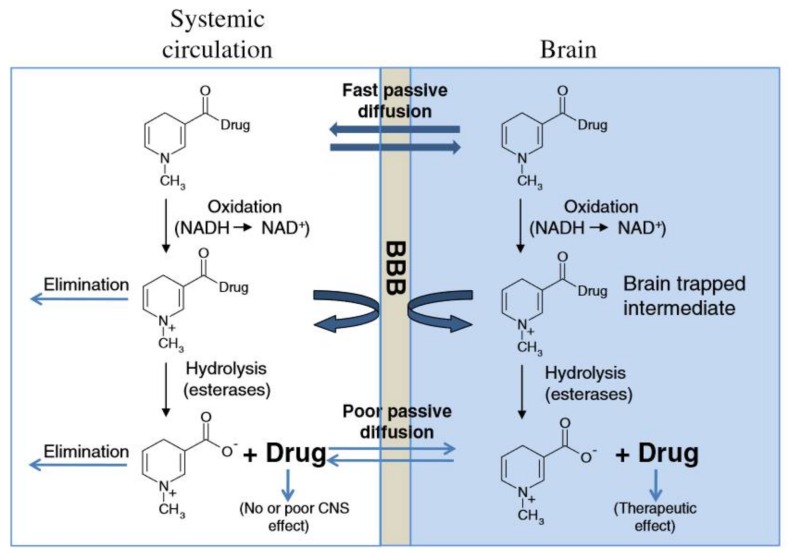
Illustration of the chemical drug delivery system. Reprinted with permission from ref. [31].

Prodrugs are different from parent drugs—they are pharmacologically inactive derivatives. It requires a transformation and activation in the body to release the active drug. They have been designed to overcome pharmaceutical and pharmacokinetic problems, because the parent drug molecule would limit the clinical usefulness. For example, drugs with –OH, −NH_2_ and −COOH terminal show the low lipophilicity, and these drugs can be esterified for generating the lipophilic drugs. After entering the CNS, the lipophilic group could be hydrolyzed for drug release. As shown in the example of Figure 2, when the hydroxy on the surface of morphine are modified by acetyl, the BBB penetration could be improved by more than 100 times.

However, the limitations for the increase of lipid solubility is obvious, few successful cases have been published. Because the P-gp on BBB could efflux the lipophilic drugs from CNS. Thus, the paclitaxel is modified for adaptation of this negative effect. The succinic acid induces in the hydroxyl of C10 position, the Tx-67 is obtained for reducing the efflux effect of P-gp and improving the BBB penetration in vitro test. Therefore, in the practical design of drugs, both these two conditions are needed to consider and evaluate.

Recently, mannitol and aromatic substances are found to show the good BBB penetration in clinical research. Mannitol was the most commonly explored osmotic agent, shrinks the endothelial cells hyperosmotically, thus opening the endothelial tight junction, which further leads to passive diffusion of large molecules across the BBB. But, the nonselective opening of BBB causes gross and uncontrolled influx of low and high molecular weight compounds and an increase of brain fluid leading to neurological toxicity, aphasia and hemiparesis that jeopardize patient safety. The clinical benefit of this method has still not been established. Although this practice is still somewhat controversial, mannitol is widely used to control elevated intracranial pressure following brain injury. Some researchers demonstrated that mannitol improved the therapeutic effects of intra-arterial transplantation of mesenchymal stem cells into brain after traumatic brain injury in an in vitro test. This effect cannot be explained solely by the increased BBB permeability induced by mannitol. But the limitation of this study is the fact that we observed transplanted mesenchymal stem cells for only up to 24 h [32]. In order to demonstrate the mannitol is useful and applicable in the clinic with certainty, a long-term assessment would be needed.

Among these aromatic substances, borneol as a simple bicyclic monoterpene with excellent properties could enhance drug permeation through the BBB, and it also widely used in traditional Chinese medicine. Tight junction proteins were transmembrane proteins containing claudins and occluding and regulating paracellular permeability and conferring blood–brain barrier function. Jin et al. found that claudin-5 and occludin was translocated from the cellmembrane to the cytoplasm at 30 min after the initiation of borneol treatment and reached peak levels at 1 h and returned to the normal pattern 8 h after initiation of treatment without significant differences in the levels of claudin-5 or occludin before or after treatment in the blood–optic nerve barrier of rats. The conclusion was that borneol exerts its permeability-enhancing effects by reversibly disassembling tight junction proteins in the BBB [33]. Yin et al. also proposed l-borneol was shown to enhance brain uptake of cisplatin (a drug with poor BBB permeability) 20 min after oral administration and gradually decreased 3 days after l-borneol administration. The results indicate that l-borneol exerts effects on BBB opening in a limited time window [34]. Recently, borneol has been investigated to enhancing penetration of rhodamine 123 entering the brain, which was associated with its regulation of the ultrastructure of brain tissues and its inhibition the expressions of Mdr1a, Mdr1b and Mrp1 transporters at the BBB [35]. The permeability-enhancing effects of borneol are closely associated with the inhibition of efflux protein function, the enhancement of transmembrane tight junction protein and predominant enhancement of vasodilatory neurotransmitters. Moreover, BBB permeability is directly and indirectly regulated by multiple neurotransmitters, particularly histamine, serotonin, *N*-methyl-d-aspartate (NMDA) and acetycholine which via nitric oxide and receptors on perivascular astrocytes and microvessel endothelial cells of the BBB. The magnitude of the increase in excitatory amino acid levels was considerably greater than that of the increase in inhibitory amino acids in the whole brain, resulting in a transient elevation in the excitation ratio (excitatory amino acids versus inhibitory amino acids) [36]. There is reason to postulate that effects of borneol in enhancing BBB permeability may be related to its temporary and predominant enhancement of vasodilatory neurotransmitters. Therefore, the possible mechanisms of borneol opening the BBB was the loosening of the endothelial tight junctions, decreasing the ABC drug efflux transporters expression, and temporary vasodilatory neurotransmitters enhancement. Therefore, borneol is believed to be an effective and promising adjuvant that can improve drug delivery to the brain.

**Figure 3:**
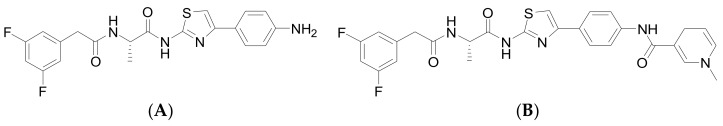
Illustration of increasing the cerebral concentration of γ-secretase inhibitors by chemical drug delivery systems. (**A**) The concentration of drugs in brain 240 ng/g; (**B**) the concentration of drugs in brain 345 ng/g. Reprinted with permission from ref. [39].

On the other hand, chemical drug delivery systems (CDDS) have been found to be a good way to strengthen BBB penetration. CDDS are composed of drugs and bioremovable targeting materials [37,38]. These components are connected as an inactive drug precursor by a covalent bond under the chemical reaction or chemical modification. After these composite nanocarriers have reached to the targeted cells, tissues or organs, the covalent bond could be cut off by the enzyme, and the active drugs are released correspondingly. Lipophilic dihydropyridine-based brain targeting of CDDS is a typical method for BBB penetration. Due to the lipophilicity of dihydropyridine, drugs connected with dihydropyridine could pass through the BBB. Once entering the brain parenchyma, the dihydropyridine is enzymatically oxidized into ionic pyridinium salt. Subsequently, the drug-pyridinium carrier is separated and the drug is sustained and slowly delivered in the brain. For instance, the γ-secretase inhibitor shows modest inhibitory activity in vitro against γ-secretase in both enzyme and cell assays. As shown in Figure 3, after combining the γ-secretase inhibitors (Figure 3A) with *N*-methyl-dihydropyridine split segments, the CDDS (Figure 3B) with the lipophilic surface was obtained. After administration in rats, compound B took two hours to reach the brain with a concentration 345 ng g^−1^ to the brain, which was about 1.5-fold of compound A (240 ng g^−1^). In short, this CDDS based on lipophilic dihydropyridine shows an effective permeability to BBB and improves the drug concentration in the brain. However, the dihydropyridine prodrug is not stable enough; this drug should be uptaked by an injection strategy.

### 3.3. Biological Methods

**Figure 4:**
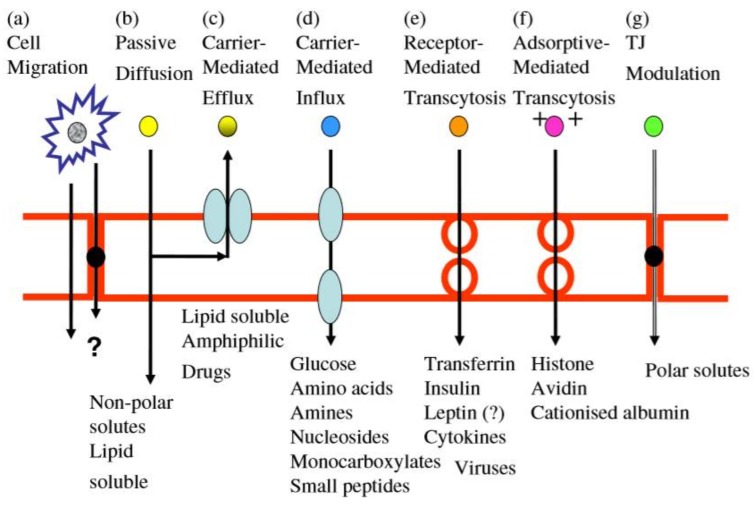
A schematic diagram of the pathway across the BBB. (a) The tight junctions for penetration of water-soluble drugs; (b) the diffusive route for lipid-soluble agents; (c) the carrier-mediated transcytosis (CMT) and (d) active efflux transcytosis; (e) specific receptor-mediated transcytosis (RMT); (f) adsorptive-mediated transcytosis (AMT); (g) tight junction (TJ) modulation. Reprinted with permission from ref. [45].

Biologically, large molecular weight solutes, such as proteins and peptides, could cross the BBB by endocytosis mechanisms of macromolecules. Although the majority of large bloodborne molecules were physically prevented from entering the brain by the presence of the tight junctions in the BBB; specific and some nonspecific transcytotic mechanisms still exist to transport a variety of large molecules and complexes across the BBB. As shown in Figure 4, there are seven routes for BBB penetration. Route (a) shows the leukocytes across the BBB adjacent to, or by modifying, the tight junctions. Route (b) is the transcellular lipophilic pathway for the penetration of lipid agents. These two routes are passive penetrations of the BBB. While in Route (c) to (g), the active efflux carriers, carrier-mediated transcytosis (CMT), receptor-mediated transcytosis (RMT), adsorptive mediated transcytosis (AMT) and tight junction (TJ) modulation are presented in Figure 4, respectively [38].

In particular, CMT works by the transport proteins on endothelium acting as carriers for the delivery of brain-necessary substances, such as glucose, amino acids, purine bases, nucleosides, and choline. It is a nonspecific manner for BBB penetration. For example, glucose carriers GLUT1 could transport glucose and other hexoses, and amino acid vehicle LAT1 could transfer several kinds of amino acids, including the amino acid drugs, and nucleotide carriers CNT2 could transfer purine nucleoside and pyrimidine nucleoside, such as uridine. The nutrient substance in blood circulation could pass the BBB into CNS by the transportation of CMT to maintain the function of CNS. If the drugs are designed as the substrate for carriers, then those drugs could be penetrated into CNS in the same way as nutrients [39]. For instance, the anticancer agent melphalan, which resembles the amino acid phenylalanine, can be transported by the LAT1 carrier [40]. Recently, the small hydrophilic drug ketoprofen, an anti-inflammatory agent that is not a substrate for LAT1, is chemically bound via an ester linkage to the phenolic hydroxyl group of the amino acid tyrosine, where an LAT1 substrate is formed, and this substrate can be recognized by the LAT1 transporter. This method opens a new possibility for cancer treatment by small molecular drugs. However, when transport by amino acid transporters, the a-amino and a-carboxyl groups of the amino acid must be free, and therefore this method cannot be used for conjugation.

RMT is expected to be vesicular-based systems that carry their macromolecule content across the endothelial cells. RMT requires receptor binding of a ligand, and it can transport a variety of macromolecules and proteins, such as peptides, insulin, and transferrin, to across the cerebral endothelium (transcytosis) [41,42]. So far, the receptors with a high expression on the endothelial cells of BBB comprise not only the insulin receptor, transferrin receptor (TfR) and low-density lipoprotein receptor, but also other receptors under exploration. Among all these receptors, the most extensively studied receptor is TfR. It is expressed on highly proliferating cells, such as cancer cells and at much higher levels on endothelial cells of the BBB than on endothelial cells at other locations within the body. For instance, the diphtheria toxin (DT) is a single polypeptide; it is also a target drug for tumor-killing. After connection with the receptors, the BBB penetrability of DT into CNS is improved, and this method is applied at the clinical stage [43]. Angiopep-2, as one of the peptides, shows enhanced transcytosis across a brain endothelial monolayer system. In recent research, a novel angiopep-2-paclitaxel conjugate is designed to improve delivery of paclitaxel across the BBB. Such a finding is consistent with the proposed BBB transport mechanism for angiopep-2 as a drug delivery vector and the beginning of the phase III clinical trial.

AMT has provided another way for brain delivery of medicines across the BBB. The surface of cerebral endothelial cells is negatively charged, thus the proteins positively charged surface could absorb on the surface of cerebral endothelial cells by AMT. It requires the interaction of a ligand with moieties expressed at the luminal surface of cerebral endothelial cells. A lot of molecules including various cationic proteins could penetrate into the brain via AMT, such as histone, protamine, and avidin. These proteins are the polycationic proteins, and they can pass through BBB without any other associated agents. Moreover, mixing the polycationic proteins or amino acid (such as protamine, poly-l-lysine) with other proteins could greatly increase the permeability of these proteins across endothelial cells. For example, native plasma proteins, such as albumin, show poor transportation in BBB. But after modification with hexamethylenediamine in preclinical studies, their brain uptake is improved greatly by employing the adsorptive-mediated endocytosis and transcytosis mechanism [44].

**Figure 5:**
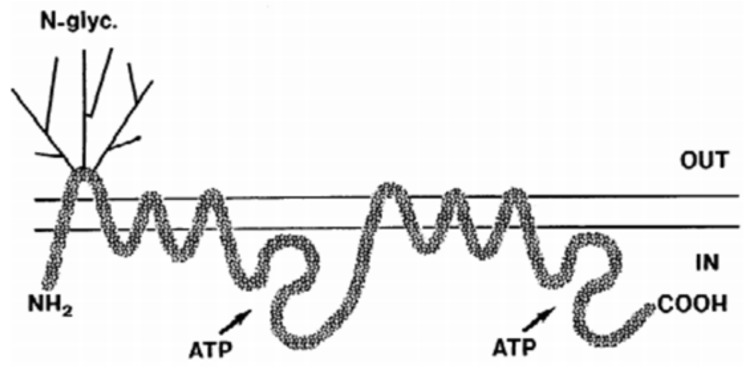
Schematic of the transmembrane structural organization of human P-glycoprotein (P-gp, 1280 amino acids long). Reprinted with permission from ref. [51].

However, active efflux transporters (AET) were important for the endothelial cells. AET could accumulate high concentrations of molecules in the cell, whereby the transporters, such as P-glycoprotein (P-gp), multidrug-resistant protein, multidrug resistance-associated protein, and peptide transport system-1, could participate in the efflux of therapeutic agents (such as vinca alkaloids, cyclosporin A and AZT) into the blood flow as soon as internalized by cells of the BBB. For example, the membrane-bound transporter P-gp acts as an efflux pump in the brain, and the transmembrane structural organization of P-gp is presented in Figure 5. Emerging evidence suggests that the P-gp may restrict the uptake of several antidepressants into the brain, thus causing the poor success rate of current antidepressant therapies. For example, first-generation P-gp modulators (i.e., verapamil, cyclosporine A, tamoxifen) have low binding affinities that require use of high doses that were proven to be toxic and have failed clinically [46]. Second-generation P-gp of valspodar inhibitors (PSC 833) and dexverapamil were developed to provide a lower systemic toxicity. However, they also inhibit theCYP3A4 metabolizing enzymes and other ABC transporters, and can dramatically increase plasma drug concentrations [47]. For example, valspodar inhibits the CYP3A4-mediated metabolism of paclitaxel, leading to potentially unsafe serum concentrations. Third-generation inhibitors with the highest specificity and fewer adverse effects were therefore developed. Most notable examples include laniquidar (R101933), tariquidar (XR9576), and elacridar (GF120918/GG918). M. Bauer et al. reported on tariquidar as a near-complete inhibitor of P-glycoprotein by performing positron emission tomography (PET) scans with the Pgp substrate (R)-[^11^C] verapamil in the BBB of five healthy human volunteers [48]. Also, N. Tournier et al. has found that combined with the P-gp inhibitor of elacridar could increase the brain uptake of erlotinib in nonhuman primates [49]. Despite the success of the third-generation P-gp inhibitors in preclinical studies, most of them have largely failed in clinical trials and did not improve therapeutic efficacy of drugs. Therefore, fourth-generation P-gp inhibitors have been suggested as a novel strategy by using natural products, peptidomemetics, and dual-targeting approaches (i.e., Lamellarin I), which are scarcely studied and are still under investigation [50]. With the development of P-gp inhibitors, the risk of adverse events may outweigh the potential therapeutic benefits, which should be a significant consideration.

### 3.4. Nanoparticles Drug-Delivery System Method

In recent year, nanoparticles become the new transports for penetrating the BBB. These nanoparticles with a size range of 10 to 1000 nm are composed of microemulsion, polymer, and inorganic materials. In recent years, many drugs have been successfully delivered by nanoparticles to cross the blood–brain barrier, such as 6 peptide dalarginz, 2 peptide kytorphin, slightly camp butyl amine, cylinder sword muscarinic, adriamycin, NMDA receptor antagonists MRZ2/576 and adriamycin. Moreover, various colloidal delivery systems have been tried while various methods have been developed to improve the penetration of BBB, and the mainstream materials for increasing BBB penetration are liposomes, polymeric nanoparticles, and solid nanoparticles [52]. The specific condition is shown in Table 3. The ultrasound method was the simple approach to open the blood–brain barrier, however it is quite difficult to avoid damaging the tissue, triggering an excessive immune response, or causing cerebral hemorrhage. The method of prodrug delivery for brain has not yet been used for brain tumor chemotherapeutic drugs. More commonly, chemical modification refers to the process of making an existing drug more lipid-soluble, with the intent of increasing BBB permeability. This approach has been used extensively with antiretroviral nucleoside analogs for treating AIDS, but has not been used much with brain tumor chemotherapeutic drugs. Liposomes and sold liposome nanoparticles are new suitable delivery systems for brain. Comparing another method, the coating of the liposome has been reported to improve the brain bioavailability. It is essential to complete our knowledge base of all transport systems active at the BBB.

#### 3.4.1. Liposome 

Liposomes are lipid vesicles consisting either one or more phospholipid bilayers. They consist of an aqueous core and phospholipid bilayer shell. The core acts as a carrier for encapsulation of hydrophilic drugs, while amphiphilic and lipophilic drugs could be solubilized within the phospholipid bilayers. The liposomes have been used in drug delivery systems for a long time, and it possesses the advantages of simple preparation, low toxicity, and relatively low cost. Both lipophilic and hydrophilic drugs are easy to combine with liposomes [68,69,70]. However, high detection and clearance rates of liposomes by the reticuloendothelial system (RES) in the liver could reduce the half-life of drugs. Reducing the sizes and polyethylene glycol (PEG) modifications are the better way for improving the duration time of liposomes in the body. PEG show a lot of advantages, such as high hydrophilicity, chain flexibility, electrical neutrality and lack of functional groups—these advantages could prevent itself from interacting unnecessarily with the biological components. Moreover, it has been suggested that PEGs with a molecular weight from 2000 to 5000 g/mole are necessary to suppress plasma protein adsorption. In order to penetrate to BBB, after conjugation with specific antibodies, the targeting property to CNS of these liposomes become more effective [71,72,73,74].

**Figure 6:**
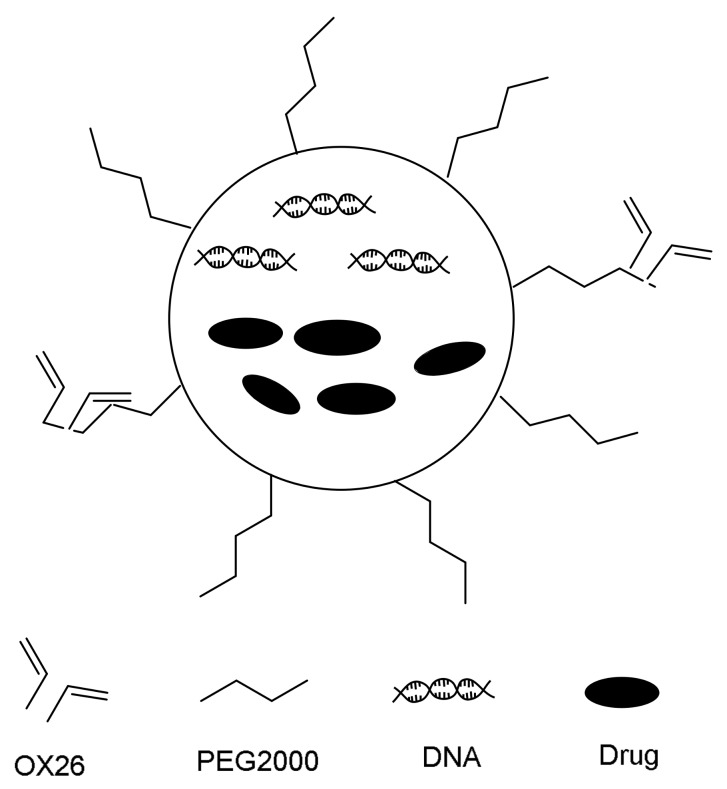
Schematic diagram of immunoliposomes (ILs).

As these DNA antibodies drug bind to a domain of the receptor that is not employed by the endogenous ligand, competition can be avoided. One of the most used peptides is the OX-26 murine monoclonal antibody (Mab), and it is directed against the transferrin receptor. This Mab connected with liposomes could improve the BBB penetration of it; this drug loading system is called immunoliposomes (ILs). The schematic diagram of the composite drug delivery is presented in Figure 6. For example, after the OX-26 are coupled with daunorubicin loaded PEG-liposomes NPs, the composite nanoparticles are crossing the BBB faster than free daunorubicin, ordinary liposomes, and PEGylated liposomes [75]. Furthermore, Gosk et al. had prepared the PEG-liposome without connection with OX-26 and OX-26-PEG-liposomes for injecting into rats by an in situ perfusion method. As a result, the uptake of OX-26-PEG-liposomes by brain endothelial cells is twice than that of pure PEG-liposome. After infusion for 15 min, the uptake rate is improved to 16 times higher than that of pure PEG-liposome via radiolabeled method [76]. This drug delivery system shows no damage to the integrity of the BBB. Therefore, the OX-26 liposomes could be used as drug transporters for BBB penetration [77,78,79,80,81,82].

#### 3.4.2. Polymeric Nanoparticles

**Figure 7:**
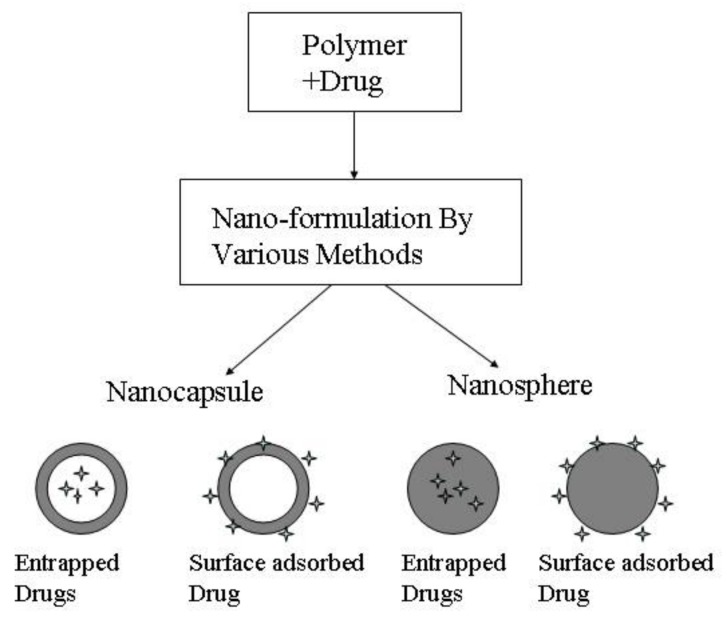
Type of polymer nanoparticles. Reprinted with permission from ref. [78].

Polymeric nanoparticles are another drug loading system for BBB penetration improvement. It represented as a solid colloidal system is composed of biocompatible one or more different polymers that have low solubility in water [83,84,85]. The specific classification is shown in Figure 7. A lot of synthetic polymers fabricated for the preparation of nanoparticles, such as poly (alkyl cyanoacrylates), polyethylene glycol (PEG), polylactic acid (PLA), poly (d,l-lactide-co-glycolate) (PLGA), etc. These prepared nanoparticles could act as carriers for drug loading by surface adsorption, covalent bond, incorporation, and encapsulation and combines with the appropriate targeting ligand that utilized biological specificity binding between antibodies, antigens, ligands and receptor to achieve active targeting [86,87,88]. However, these polymeric nanoparticles are also easy to eliminate because of opsonization by RES cells. Thus, the modification of these nanoparticles is a necessary procedure for improving the circulation of nanoparticles in the blood. For example, Krueger performed an in vivo experiment that confirmed the polybutylcyanoacrylate nanoparticles integrating with polysorbate 80 improved the opioid peptide dalargin delivery to brain. As a contrast, without the modification of the polymer, the delivery ability of H3-dalargin across the BBB obviously decreased. The mechanism to explain this phenomenon may be the interaction between nanoparticles and receptors of low density lipoprotein (LDL) on the surface of brain endothelial cells. The polysorbate 80 could accelerate the endogenic LDLs from the plasma interaction with the brain endotheliocytes by the Trojan horse principle [89]. Moreover, PLA is a polymer based on lactic acid as a main polymer, which is a degradable polymer with good biocompatibility and safety and is nontoxic. However, due to the presence of the main-chain side methyl of PLA, the polymer is less hydrophilic. The hydrophilic PEG is induced to change this problem. For instance, Xia and co-workers proposed that cell-penetrating peptides (CPPs) were decorated with PEG-PLA nanoparticles to obtain satisfactory pharmacokinetic and biodistribution profiles for brain drug delivery in cells. The prepared penetrating-NP shows a particle size of 100 nm. The better experiment shows that PEG-linked liposomes could, through brain through receptor-mediated interactions, carry small-molecule drugs to reach the brain without damaging the blood–brain barrier [90].

PLGA is the polymer composed of lactide and glycolate. It is similar to PLA and could be used for therapy of Alzheimer’s disease. Stefanie and coworkers presented that the influence of alterations in the composition of the PLGA nanoparticles could influence the antitumor effects of adriamycin (DOX) in the rat glioblastoma model. This result is obtained by employing histological and immunohistochemically methods with the objective to enable a further optimization of this delivery system and a better BBB penetration [91,92].

#### 3.4.3. Solid Lipid Nanoparticles

In addition, solid nanoparticles (SLNs) were another promising CNS drug-delivery system because of excellent hydrophobicity covered with phospholipid layer. The solid nanoparticle is covered by a phospholipid layer, and the targeting ligands are induced into these nanoparticles for enhancing the penetrability of BBB by receptor-mediated transcytosis or inhibition of efflux transport. By comparing with polymer nanoparticles and liposomes, these solid nanoparticles show higher capacity for drug loading, greater stability, lower cytotoxicity, controlled release properties and relatively lower cost. SLNs may carry both lipophilic and hydrophilic drugs. A lot of substances are delivered into the brain by these SLNs, such as anticancer drugs camptothecin, docetaxel, small interfering RNA, idebenone, apomorphine and risperidone [93,94,95].

For example, Yang had assessed in vivo specific drug targeting performance of the camptothecin-loaded SLNs to prolong drug release via the oral route. Under the detection of reversed-phase, high-performance liquid chromatography with a fluorescence detector, the concentrations of camptothecin in mice was determined in reticuloendothelial cells containing organs as well as in organs containing no reticuloendothelial cells [96]. Loureiro had fabricated solid lipid nanoparticles (SLNs) functionalized with OX26, work as a possible carrier to transport the extract to target the BBB in human brain-like endothelial cells. Experiments on human brain-like endothelial cells show that the cellular uptake of the OX26 SLNs is substantially more efficient than that of normal SLNs and SLNs functionalized with an unspecific antibody. Stability studies were performed to assess the use of these SLNs as a promising future drug delivery system, and the results showed that the nanoparticles are stable for a minimum period of one month [97]. Moreover, Koziara suggested that the brain uptake of paclitaxel was significantly increased using emulsifying SLNs delivery system. Possible mechanisms include (1) the limited access of drug to P-gp by nanoparticle entrapment; (2) modulation of BBB p-gp by the surfactant and (3) opening of the BBB in presence of nanoparticles [98].

#### 3.4.4. Magnetic Nanoparticles

The magnetic nanoparticles (magnetite, i.e., Fe_3_O_4_) are materials that can control drug delivery via an external magnetic field, and the penetrated ability in biofilms and bacteria for these Fe_3_O_4_ nanoparticles could be further improved by functionalizing their surface with molecules or chemicals. As some in vivo experiments demonstrated, surface-functionalized Fe_3_O_4_ are used to enhance magnetic resonance imaging contrast, tissue repair ability, drug delivery performance and cell separation efficacy. After injecting Fe_3_O_4_ surrounding a biofilm, an external magnet field is used to draw the nanoparticles into the biofilm to disrupt bacteria functions. Recently, many studies also demonstrate that the antibiotics coated magnetic particles are prepared as an alternative method for drug delivery. Albeit such approach is still relying on the antibiotics, the antibacterial activity of these composited nanoparticles is still improved increasingly [99]. Additionally, Kong et al. had successfully created MNPs with the size of 100 nm, which provided improved BBB crossing. With combined fluorophore, the position of MNPs could be directly tracked in the brain parenchyma of normal mice, which also allow cellular-level by confocal microscopy. The magnetic nanoparticles could target for extravasation and assembly by apply magnetic field which could be used for crossing BBB drug delivery to treatment of various CNS diseases [100,101,102].

#### 3.4.5. Novel Nanoscale Entities

Many novel nanoscale entities could be employed as efficient drug delivery vectors for BBB penetration. Namely, there exists two strategies available for researchers and professionals: structure-driven nanoscale entities and size-driven nanoscale entities. The former route designs, constructs and fabricates hollow, porous (various nanoparticles) and flexible, even soft (peptides, gene, DNA, bio-dendrimers etc) structures in a nanoscale dimension that are biologically compatible with BBB penetration, while the latter route does utilize the size selection principle to prepare nanoscale entities that are also biologically compatible with BBB penetration, including CNT-based drug delivery systems, cyclodextrin-based drug delivery systems and other nanosized drug delivery systems towards BBB penetration improvements [103,104,105,106,107,108,109,110].

A promising way to promote BBB penetration and deliver drugs to the targets within the CNS is the employment of nanoparticles. The nanoparticles drug delivery system has an excellent property itself, which not only can increase the concentration and prolong the residence time of the drug in the brain. Nanoparticles as drug delivery vectors brain have a high practical value and research value. Some drugs with nanoparticles as a carrier have entered the stage of clinical stage, but most of the research still in animal testing. Hopefully, some lipid nanoparticles already have entered the clinical important prerequisite, which is expected to be put into mass production. There is still many problems in brain-targeting nanoscale drug delivery systems: (1) tissue compatibility, security, and quality control; (2) NP mechanism of drug brain delivery release; (3) The factors affecting the NP into the brain, such as the material, size, shape, preparation, surface modification and so on. Inevitably, these strategies will take priority and drive future development for improving drug permeability of BBB.

## 4. Future Challenges of Delivery Drug across BBB to Brain

With the developed method of drug delivery across the BBB to treat the brain disease, the current lack of in vivo validation of the BBB and lack of controlled clinical trials that address CNS drug delivery hinder the advancement of brain drug delivery. The transferrin, insulin and LRP receptors have an important impact on the biological, scavenger and signaling functions for blood–brain drug delivery, and many researchers have carried out successful clinical development of technologies based therein [111]. In the past year, angiopep-2 conjugated to paclitaxel (ANG1005/GRN1005) and glutathione pegylated liposomal doxorubicin (2B3-101) have been or are currently being investigated in clinical studies. In addition, ANG1005 (recently renamed as GRN1005) has been recognized as safe for patients of brain metastases or glioma in clinical phase I/II studies [112]. However, because this receptor is the membrane-bound precursor of heparin-binding epidermal growth factor (HB-EGF), prolonged targeting might influence the biological function of this growth factor. To solve this problem, the glutathione transporter seems to have the best safety profile. Another study regarding 2B3-101 as a targeting ligand to cross the BBB via glutathione transporters, was undergoing initiated clinical phase I/II study without neurobehavioral effects of the BBB [113]. Then, ligand-targeted approaches may be more effective compared to unencapsulated drugs or ligand-lacking nanomedicines, as they can improve drug delivery to the CNS via receptor-mediated transcytosis and ligand-targeted particulate nanomedicine, which will represent safe and efficacious drugs in the upcoming years.

In some drugs currently on the market, antidepressants and medications for schizophrenia and epilepsy, along with caffeine and nicotine also can readily dissolve into the lipid membranes that encase blood–brain barrier cells. Thereby, most of the small molecule combinations also have been explored to across the BBB [114]. The current FDA-approved pharmacological treatments for CNS tumors such as doxorubicin, carmustine, trastuzumab, and temozolomide have been successfully introduced across the BBB [115].

Given these challenges, blood–brain barrier research went from a cottage industry to people bringing the newest tools and approaches to bear on the subject. Finding ways to stealthily shuttle drugs across the blood brain barrier could have significant therapeutic implications. Our first hope was adhering to the criteria related to drug development from lab to clinic, but also reduce the cost.

## 5. Conclusions

With the aging of modern society’s population, brain diseases including neurodegenerative disease (such as Alzheimer’s disease and Parkinson’s disease), stroke, neuroinflammation, and neuro-oncology endanger human health tremendously. Drug delivery across the physiological barriers of the brain is the bottleneck for the treatment of CNS diseases and brain tumors. It is therefore critical to search for alternative CNS delivery routes improving the permeability of BBB to achieve effective drug concentrations in the brain. The development of BBB tests and methods, including in vivo and especially in silico methods as well as corresponding experiments, will greatly help to simplify the practical difficulties and circumvent potential ethical controversies [116,117]. Meanwhile, the release of a drug in the brain should be accurately monitored and controlled in situ or in real time. It is believed by those that study BBB transport mechanisms and the pathogenesis of the brain that the improvement of the permeability of the BBB will certainly achieve a breakthrough, resulting in great theoretical significance and economic/social benefits.

## Figures and Tables

**Table 1:** Different methods investigated to get through the BBB to deliver drugs to the brain, data from ref. [13].

Method	Advantages	Disadvantages	Reference
Direct injection	High local drug concentrations can be achieved; systemic administration avoided	Side effects; hard to control; and repeat	[14,15,16]
Transnasal delivery	Noninvasive; easy to operate and repeat; low risk	Smaller drug delivery volume; interindividual difference	[17,18,19,20]
Arterial injection of osmotic solution	High drug concentrations achieved; large clinical experience	Requires general anesthesia; side effects; hard to repeat	[21,22]
Lipidation of small molecule drug	Easy to operate; delivered to the whole brain	Applies to easily etherified drugs	[23,24,25]

**Table 2:** Reported effects of different parameters on BBB disruption via focused ultrasound and microbubbles, data from Elsevier [13].

Parameter	Effect on BBB Disruption
Pressure amplitude	Increase in BBB disruption magnitude as pressure amplitude increases; saturation at some point
Ultrasound frequency	Decrease in BBB disruption threshold as frequency decreases; some evidence of improved safety for lower frequencies
Burst length	For burst lengths, less than 10 ms, BBB disruption threshold increases and BBB disruption magnitude decreases as burst length is reduced
Pulse repetition frequency	BBB disruption magnitude increases as repetition frequency increases up to a point.
Ultrasound contrast agent dose	Magnitude of BBB disruption increases with dose
Sonication duration	Longer durations or repeated sonication increase magnitude of BBB disruption
Microbubble diameter	Disruption magnitude increased with larger microbubbles

**Table 3:** Different drugs across the blood–brain barrier.

Drug	Structure	The Method of Across the BBB	Function	Reference
*cis*-Dichlorodiamineplatinum	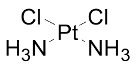	Ultrasound	alkylating agent, Antitumor Effect	[53,54]
Barbital	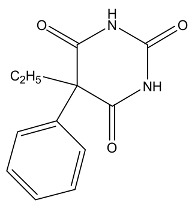	Lipophilic prodrug	Sedative-hypnotics, sedative-hypnotic, antiepileptic and anticonvulsant effect	[55,56]
5-fluorouracil	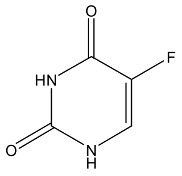	Chemical delivery systems	Antineoplastic	[57,58]
Adriamycin	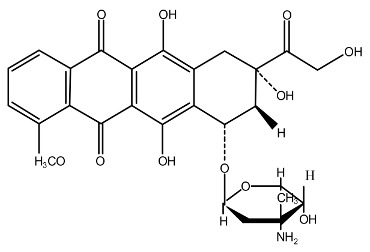	Liposomes	Antitumor antibiotics	[59,60,61]
Idebenone	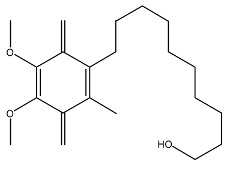	Sold liposome nanoparticles	Anti-senile dementia drug and mental symptoms drug	[62,63]
Saquinavir	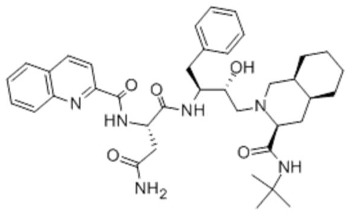	Nanocarriers	Antivirals. For selective HIV protease inhibitors.	[64]
Rivastigmine	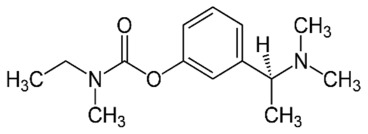	Nanocarriers	Acetylcholinesterase inhibitor	[65]
Idebenone, IDBN	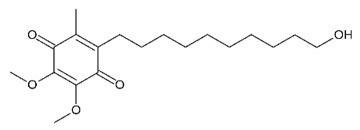	Nanocarriers	Alzheimer’s disease and cognitive defects	[66]
Curcumin	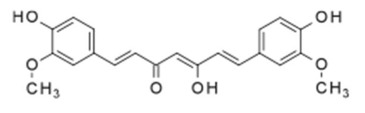	Liposomes	Antineoplastic	[67]
